# Drainage of the Metropolis

**Published:** 1857-01

**Authors:** 


### SANITARY LEGISLATION.

### DRAINAGE OF THE METROPOLIS.

Not much has been done in this direction during the past quarter, except a great deal of noise and numerous interchanges of words between the Metropolitan Board of Works and Sir Benjamin Hall. Sir Benjamin has acted with great prudence and decision. He appoints a competent committee to receive all drainage schemes, and report on them. We have in hand numerous letters and communications on various drainage plans; but as we do not see any plan that is free from objection, or is at once simple and effective, and as all the plans have been discussed to tiring point by the scientific journals, we have, *pro tem.*, but little to say. Mr. Nicholas, of Wandsworth, has placed before us some very sound views on the general question of a drainage scheme. He sees the necessity of commencing the flushing process from each house. To leave our drainage and sewerage in the present state, and to be content to open only one immense general sewer for the reception of the tributary sewerage now thrown into the Thames, must, in a sanitary point of view, argues Mr. Nicholas, be a failure. We believe he is right.

